# Neural correlates of aggression in personality disorders from the perspective of DSM-5 maladaptive traits: a systematic review

**DOI:** 10.1038/s41398-023-02612-1

**Published:** 2023-10-26

**Authors:** Nathan J. Kolla, John Tully, Katja Bertsch

**Affiliations:** 1 Department of Psychiatry, University of Toronto, Toronto, ON Canada; 2 Centre for Addiction and Mental Health, Toronto, ON Canada; 3 Department of Psychiatry, University of Saskatchewan, Saskatoon, SK Canada; 4 Academic Unit of Mental Health and Clinical Neurosciences, School of Medicine, University of Nottingham, Nottingham, United Kingdom; 5 Department of Psychology, Ludwig-Maximilians-University, Munich, Germany; 6 NeuroImagine Core Unit Munich (NICUM), University Hospital LMU, Munich, Germany; 7 Department of General Psychiatry, Center for Psychosocial Medicine, Heidelberg University, Heidelberg, Germany

**Keywords:** Neuroscience, Diseases, Biomarkers

## Abstract

The fifth edition of the Diagnostic and Statistical Manual of Mental Disorders (DSM-5), published in 2013, includes an alternative model of personality disorders (AMPD) focusing on a maladaptive trait model utilized to diagnose several personality disorders. Borderline personality disorder (BPD) and antisocial personality disorder (ASPD) are two conditions categorized by AMPD that exhibit high rates of violence and aggression. Several of the traits outlined in the AMPD, including hostility, impulsivity, risk-taking, and callousness, have been previously linked to aggression in BPD and ASPD. However, to the best of our knowledge, there has never been a synthesis of neuroimaging studies that have investigated links between these traits and aggression in BPD and ASPD. To overcome this gap, we conducted a systematic review under the PRISMA framework to locate neuroimaging articles published since the release of AMPD linking trait anger/hostility, impulsivity, risk-taking, and callousness to aggression in BPD and ASPD. Key findings included the following: i) anger/hostility, associated with alterations in the interplay between prefrontal and subcortical regions (primarily the amygdala), may be a common factor explaining aggressive reactions to response to interpersonal threat or provocation; ii) alterations of fronto-temporal-limbic regions and serotonergic and endocannabinoid signaling systems may link impulsivity to aggression in BPD and ASPD; iii) weaker cortico-striatal connectivity could relate to greater risk taking and greater proclivity for violence. Insufficient evidence from neuroimaging articles was discerned to describe a relationship between callousness and aggression. Overall, results of this review reveal a relative paucity of neuroimaging studies examining AMPD traits relevant to aggression in BPD and ASPD. In addition to encouraging further investigation of neuroimaging markers of AMPD traits linked to aggression, we recommend multi-methodological designs, including the incorporation of other biomarkers, such as hormones and indices of physiological arousal, to fully expand our understanding of aggression in BPD and ASPD.

## Introduction

Aggression is a common clinical feature of antisocial personality disorder (ASPD; [1]) and borderline personality disorder (BPD; [2]). It contributes to detrimental outcomes in both conditions, with much higher rates of violent offending in both ASPD and BPD [1, 3, 4]. Comorbidity of these conditions is common [5, 6] and associated with increased rates of aggression and violence [3, 7, 8]. Evidence for treatment options targeted at reducing aggression in ASPD [9, 10] and BPD [11] is poor, and development of effective treatments is hampered by a limited understanding of the mechanistic basis of aggression in these conditions. One pathway to a better understanding of such mechanisms is the use of neuroimaging, which can detect structural, functional, and neurochemical abnormalities and link these to subtypes of aggressive behavior [12, 13], thereby identifying potential therapeutic targets.

Most of the initial neuroimaging research in BPD and ASPD failed to consider the potential link between specific personality disorder traits and neural markers of aggression. However, early in the last decade, a shift towards a transdiagnostic, trait-based approach was reflected by the inclusion of an alternative model of personality disorders (AMPD) in Section III of the fifth edition of the Diagnostic and Statistical Manual of Mental Disorders (DSM-5). This demonstrated that while the field was not yet prepared to abandon categorical diagnoses, a framework for researching the trait-based nature of these disorders had emerged (see Box 1). To advance these approaches, neuroimaging research would optimally link behavioral outcomes, such as aggression, to core traits that overlap across two or more personality disorders. Since the publication of DSM-5 in 2013, there have been a number of reviews examining the neurobiological correlates of aggression in BPD [14–17], ASPD [18, 19], and personality disorders as a group [20], as well as in non-clinical samples [21]. However, the degree to which DSM-5 has influenced empirical approaches remains unclear. Importantly, no study has systematically appraised the neuroimaging literature linking specific maladaptive personality traits to neural metrics of aggression within these important clinical disorders.

We, therefore, sought to systematically review the literature since 2013, using a trait-based approach. As previous evidence suggests that anger/hostility [22–26], impulsivity [23, 25–27], risk taking [22], and callousness/lack of empathy [22, 28], are linked to aggression in BPD and/or ASPD, we focused on studies linking measures of one or more of these four traits to metrics of aggression in BPD and/or ASPD. As ASPD is present in up to 80% of prison samples [29], we also considered studies in samples of incarcerated offenders, in which the Psychopathy Checklist-Revised [29] is commonly used as a measure of the degree of antisociality. As there is significant overlap between these conditions and intermittent explosive disorder (IED), we discuss this condition separately (see Box 2).

Box 1 AMPD personality disorder As noted above, DSM-5 contains an alternative model of personality disorders (AMPD) in Section III that was introduced as a candidate to replace categorical classification of personality disorders (PDs) [93]. The dimensional core of AMPD comprises two criteria. Criterion A involves general, personality-based impairment related to the self and interpersonal functioning and is the basis for deciding whether an individual qualifies for a PD. Criterion B includes an evaluation of pathological personality traits, mainly operationalized using the Personality Inventory for DSM-5 (PID-5) [94] that captures extreme, maladaptive variants of Five-Factor Model (FFM) traits [91]. The AMPD traits include 25 maladaptive facets grouped into five higher-order broad domains: negative affectivity, detachment, psychoticism, antagonism, and disinhibition [94]. The AMPA model permits clinicians to draw upon six traditional PD types, including borderline, antisocial, obsessive-compulsive, schizotypal, avoidant, and narcissistic. To diagnose ASPD, according to AMPD, six or more of the following pathological personality traits must be present: manipulativeness, callousness, deceitfulness, hostility, risk taking, impulsivity, and irresponsibility. Individuals with ASPD may also be classified “with psychopathic features.” To diagnose BPD, according to AMPD, four of the following seven personality traits must be present, at least one of which must be impulsivity, risk taking, or hostility: emotional lability, anxiousness, separation insecurity, depressivity, impulsivity, risk taking, and hostility. See Table 1.

**Table 1:** AMPD traits examined in our study.

AMPD trait	Part of borderline personality disorder	Part of antisocial personality disorder	Criterion B pathological personality trait domain(s)^a^	Description
Hostility (/anger)	Y	Y	Negative Affectivity, Antagonism	Persistent or frequent angry feelings; anger or irritability in response to minor slights and insults; mean, nasty, or vengeful behavior.
Impulsivity	Y	Y	Disinhibition	Acting on the spur of the moment in response to immediate stimuli; acting on a momentary basis without a plan or consideration of outcomes; difficulty establishing and following plans; a sense of urgency and self-harming behavior under emotional distress.
Risk-taking	Y	Y	Disinhibition	Engagement in dangerous, risky, and potentially self-damaging activities, unnecessarily and without regard to consequences; lack of concern for one’s limitations and denial of the reality of personal danger; reckless pursuit of goals regardless of the level of risk involved.
Callousness (/lack of empathy)	N	Y	Antagonism	Lack of concern for the feelings or problems of others; lack of guilt or remorse about the negative or harmful effects of one’s actions on others.

^a^Criterion B: pathological personality traits are divided into 5 broad domains: negative affectivity, detachment, antagonism, disinhibition, and psychoticism.

The AMPD was originally designed to mitigate shortcomings of the current diagnostic approach to PDs, for example, poor reliability, heterogeneous clinical presentations, and high rates of comorbidity [95]. A substantial body of research supports the dimensional trait model [96–98]. Moreover, meta-analysis strongly suggests that dimensional models of personality pathology, such as the AMPD, are perceived by clinicians as more advantageous than a categorical approach [99]. Despite a fast-growing evidence base, only recently has neurobiological investigations of the AMPD begun to emerge. For example, an investigation examining biobehavioral risk for externalizing problems in adults reported that trait scales of the PID-5 captured by the disinhibition domain did not accord well with a neurophysiological externalizing factor indexed by P3 brain responses but that an alternative trait-scale drawing upon the impulsivity, irresponsibility, and distractibility facets of the disinhibition domain coupled with the hostility facet of the negative affect domain interfaced effectively [62]. To the best of our knowledge, there has not been a synthesis of neuroimaging studies that have investigated links between PID-5 facets hypothesized to relate to aggression in personality disorder populations. This omission is noteworthy, since some authors have written that the utility of AMPD could be strengthened by more neurobiological research [100].

In deciding on which traits to focus on in relation to aggression, we were guided by a recent publication that sought to compare PID-5 associations with self-report and collateral reports of aggressive behavior, one of the few studies to examine PID-5 associations in relation to aggression [22]. This study reported that in a sample of outpatients with personality disorders, the most important PID-5 predictors of aggression were hostility, risk taking, and callousness. Therefore, we focused on these three traits, in addition to impulsivity, to conduct a systematic review linking these traits to aggression in BPD and ASPD.

Box 2 Intermittent explosive disorder and overlap with BPD and ASPD Intermittent explosive disorder (IED) captures individuals with recurrent, problematic, and impulsive aggression [101] and is highly relevant to our discussion because approximately half of this population has a comorbid personality disorder [102]. ASPD and BPD are the most common comorbid personality disorders, and the combination of IED with either ASPD or BPD is associated with the highest level of aggressive behavior, though not necessarily increased impulsivity [102]. IED is often comorbid with other psychiatric disorders, such as current bipolar disorder, depression, anxiety disorders, substance use disorders, and posttraumatic stress disorder, and aggression scores are typically higher in comorbid groups [92]. Several neuroimaging investigations have begun to delineate the neural correlates of IED and impulsive aggression. One sMRI study of 57 IED patients (43.9% with comorbid BPD, 17.5% with comorbid ASPD, and 17.5% with comorbid psychopathic personality disorder), 58 psychiatric controls, and 53 healthy individuals found that gray matter volumes in cortical (OFC, mPFC, ACC) and subcortical (amygdala, uncus, insula) structures were lowest in the IED group [103]. Across all participants, gray matter volumes were inversely related with a composite dimensional measure of aggression (e.g., Aggression score from the Life History of Aggression interview [104] and the aggression score from the Buss–Perry Aggression questionnaire [105]), although the presence of IED (as opposed to aggression score) better accounted for the variability of frontolimbic gray matter volume values across subjects, consistent with a role for faulty frontolimbic circuitry in the pathophysiology of impulsive aggression [106]. To test the hypothesis that IED is associated with abnormalities in white matter integrity, white matter diffusion anisotropy was compared between 42 IED patients (20% with comorbid ASPD and 45% with comorbid BPD), 50 psychiatric controls, and 40 healthy controls using tract-based spatial statistics [107]. Results revealed that IED was associated with lower fractional anisotropy in two clusters located in the superior longitudinal fasciculus compared with the other groups [108]. Moreover, impulsive aggression and BPD, but not ASPD or psychopathy, was associated with lower fractional anisotropy in the two clusters within the superior longitudinal fasciculus. These findings provide evidence for disruption of long-range white matter tracts between frontal and temporoparietal regions in IED, BPD, and impulsive aggression, which may suggest a role for genetic factors, since the fractional anisotropy of the superior longitudinal fasciculus has been found to be moderately heritable [109]. In summary, IED epitomizes pathological impulsive aggression and is often comorbid with BPD and ASPD. Neuroimaging studies of IED shed light on potential neurobiological abnormalities underpinning impulsive aggression, with findings having relevance for all psychiatric disorders presenting with high levels of impulsive aggression.

## Methods

A systematic literature search was undertaken according to the Preferred Reporting Items for Systematic Reviews and Meta-Analyses (PRISMA) guide. The search was restricted to papers published between 2013 and 2022 and the following databases were searched in October 2022: MEDLINE® ALL, Embase (via Ovid), APA PsycInfo®, EBSCO CINAHL, and APA PsycArticles® (via ProQuest). We searched for papers from 2013 onwards, as this was the date when DSM-5 was published and the AMPD was first introduced.

The search was conducted by an information specialist (NT, see Acknowledgements), using a combination of free text terms (searching the title and abstract) and relevant controlled vocabulary headings customized for each database, as well as advanced search syntax (truncation, Boolean logic AND/OR, and proximity searching), to ensure all relevant studies were identified. The search terms included the following themes, with synonyms to describe each: borderline personality disorder or emotionally unstable personality disorder or antisocial personality disorder or psychopathy, traits (hostility, impulsivity, risk- taking or callousness), aggression, and neuroimaging.

Studies were initially included if they were (1) published as a peer-reviewed article with original data in adult samples using structural MRI (sMRI), functional MRI (fMRI, including measures of functional connectivity), positron emission tomography (PET), magnetic resonance spectroscopy (MRS), and diffusion tensor imaging (DTI); (2) included individuals with BPD and/or ASPD (with or without psychopathy), defined using standardized classification tools (DSM or ICD criteria for BPD and ASPD) *or* included incarcerated offenders with a Psychopathy Checklist- Revised (PCL-R) score for psychopathy; (3) included a quantifiable, standardized metric of at least one of the following: risk-taking, impulsivity, hostility, or callousness/lack of empathy; *and* (4) investigated the link between at least one of these traits and a metric of aggression using a neuroimaging technique. Exclusion criteria were the following: 1) review articles; 2) dissertations; 3) letters to the editor; 4) opinion articles; 5) editorials; and 6) case reports or case series.

**Fig. 1:**
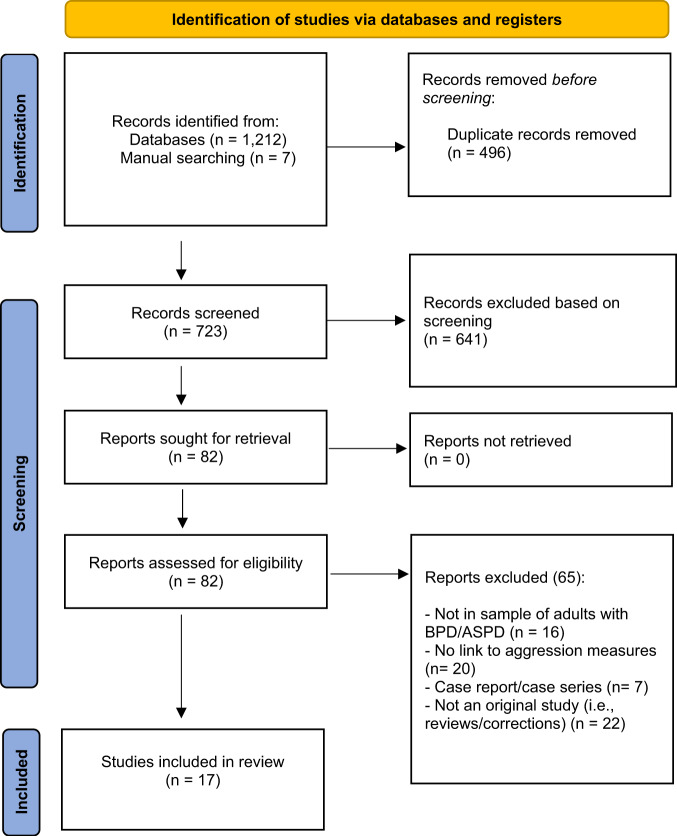
PRISMA flow diagram of the search strategy. Identification, screening, and inclusion of articles in our systematic review.

Our PRISMA diagram (see Fig. 1) illustrates our search strategy. Our initial search strategy identified 1,212 records. We then supplemented the search by manual and bibliographic cross referencing and by examining previous systematic reviews and meta-analyses to identify potentially missed studies. This strategy revealed 7 additional records. Once duplicates had been removed, we had 723 records. We screened out 641 records based on titles and abstracts. We retrieved the remaining 82 studies to assess eligibility. We included 17 studies based on the search strategy. The search screening and data extraction were completed independently by three separate researchers: NJK, JT, and KB. Disagreements were discussed and finalized by consensus vote. For each extracted article, we recorded the study author, trait being examined, neuroimaging technique, metric of aggression, sex composition of the sample, personality disorder diagnosis, and main neuroimaging findings. See Table 2.

**Table 2:** All included studies.

Author	AMPD trait	Measure of aggression	Composition of sample	Main findings
Bertsch et al. [33]	Anger/Hostility	STAXI Anger Out	15 male anger-prone patients with BPD, 25 healthy male controls	BPD showed reduced ventrolateral PFC activation during emotional action control (*t* = 3.04, *p* = 0.05). Anger out was negatively related to ventro- (*t* = 3.04, *p* = 0.050) and dorsolateral PFC activation (L *t* = 3.75, *p* = 0.009, R *t* = 4.34, *p* = 0.002), while it was positively related to amygdala activity in BPD (*t* = 5.15, *p* < 0.001).
Bertsch et al. [30]	Anger/Hostility	OAS-M aggressive behavior & aggressive behavior in experiment (STAP)	48 female patients with BPD, 28 healthy female controls	BPD showed less differentiation between trials with angry vs. neutral looking opponent in aggressive behavior, amygdala (L *t* = 4.39, pFWE < 0.001, R *t* = 3.17, pFWE = 0.028) and OFC (*t* = 3.74, pFWE = 0.005) response; amygdala response to neutral faces in BPD was related to aggressive behavior in experiment (L *r* = 0.25, *p* = 0.082, R *r* = 0.32, *p* = 0.025).
Neukel et al. [38]	Anger/Hostility	OAS-M	33 aggressive patients with BPD (20 receiving specific anti-aggression group psychotherapy; 13 patients non-specific control psychotherapy), 25 healthy controls	Decrease in amygdala activation in response to facial stimuli after anti-aggression therapy (p_FWE_ = 0.046), whereas a non-significant increase in amygdala activation was found after non-specific psychotherapy. Furthermore, in the anti-aggressive therapy group, connectivity between amygdala and dorsomedial prefrontal cortex increased from pre- to post-treatment compared to the non-specific psychotherapy group.
da-Cunha-Bang et al. [40]	Anger/Hostility	BPAQ; STAXI; aggressive behavior in experiment (PSAP)	18 male incarcerated violent offenders with PD (14 with ASPD, 2 with BPD), 26 male non-offenders	Violent offenders behaved more aggressively in the PSAP and showed higher response to provocation in amygdala (*t* = 2.5, df = 32.9, *p* = 0.02) and striatum (*t* = 2.5, df = 26.5, *p* = 0.02) and reduced amygdala-PFC (*k* = 527 voxels, *z* = 3.78) and striato-PFC connectivity (*k* = 349 voxels, [−10,40, −8], *z* = 3.69, *p*-value not provided); amygdala reactivity to provocation was positively related to task-related behavior in offenders SE: 13.4, CI: −27.3;25.5).
Cunha-Bang et al. [39]	Anger/Hostility	Latent factor of reactive aggression generated from: BPAQ, STAXI, angry hostility and impulsivity from NEO-PI-R; BIS trait impulsivity; PPI-R self-centered impulsivity.	19 male incarcerated violent offenders with PD (14 with ASPD, 2 with BPD), 28 male non-offenders	The latent factor of aggression was related to amygdala reactivity to fearful (ß = 3.8, CI [1.5; 6.1], *p* = 0.001) but not angry (ß = 0.02, CI [−3.2; 3.3], *p* = 0.9) faces. Offenders did not differ from healthy controls in amygdala response.
Herpertz et al. [37]	Anger/Hostility	STAXI	33 anger-prone female patients with BPD, 23 anger-prone male patients with BPD 30 healthy female controls, 26 healthy male controls	Male BPD show higher activation in left amygdala than female BPD (*t* ≥ 3.89, pFWE < 0.05) and male HC (*t* ≥ 4.69, pFWE < 0.05) to scripts of rejection and aggression; male BPD show higher lateral orbitofrontal (*t* = 4.30, pFWE < 0.05) and dorsolateral prefrontal (*t* = 4.48, pFWE < 0.05) activity compared to male HC and female BPD; positive connectivity between amygdala and posterior middle cingulate cortex in female BPD, but negative connectivity in male BPD (*t* = 3.99, *p* < 0.01); negative modulatory effects of trait anger on amygdala dorsolateral prefrontal (*t* = 4.94, *p* < 0.001) and amygdala lateral OFC coupling in male BPD (*t* = 6.00, *p* < 0.001); in female BPD: trait anger modulated positively dorsolateral prefrontal-amygdala coupling; trait anger modulated positively connectivity of left amygdala and posterior thalamus in men but not in female BPD (*t* = 5.62, *p* < 0.001).
Siep et al. [42]	Anger/Hostility	BPAQ, RPQ	18 violent male offenders (11 with ASPD, 3 with BPD, 3 with paranoid PD), 18 healthy male controls	Self-reported anger was positively related to medial prefrontal activity pre-task (*r* = 0.36, *p* < 0.05) and increased during the emotional task in both groups; significant decrease in amygdala-medial prefrontal connectivity in offenders and increase in non-offenders after the task. Increase in connectivity between amygdala and (para) limbic regions in offenders and decrease in non-offenders after task.
Tonnaer et al. [41]	Anger/Hostility	BPAQ, RPQ	16 violent male offenders (9 with ASPD, 3 with BPD, 4 with other PDs), 18 healthy male controls	Increased ventrolateral prefrontal activity during anger engagement in offenders (*t* = 2.31, *p* = 0.027), decreased dorsolateral and ventrolateral prefrontal activity during anger distraction (*t* = −3.15, *p* < 0.001 and *t* = −2.55, *p* = 0.022); less activity in amygdala during anger regulation is related to aggression in RPQ (*r* = −0.45, *p* = 0.007). Less activity in ventrolateral prefrontal during anger regulation is related to aggression in RPQ (*r* = −0.47, *p* = 0.005) and BPAQ (*r* = −0.45, *p* = 0.005).
Soloff et al. [46]	Impulsivity	LHA	31 female BPD; 25 female healthy control	Trait impulsivity in BPD correlated with activation in the dorsal anterior cingulate cortex (dACC, *k* = 201 voxels, *p* = 0.001), orbital frontal cortex (OFC, *k* = 140 voxels, *p* = 0.001), dorsolateral prefrontal cortex (*k* = 26 voxels, *p* = 0.001), and basal ganglia (*k* = 172 voxels, *p* = 0.001), while aggression negatively correlated with OFC (*k* = 404 voxels, *p* = 0.003), hippocampus/parahippocampus (*k* = 1041 voxels, *p* < 0.001), and basal ganglia (*k* = 379 voxels, *p* = 0.004) activation.
Soloff et al. (2014a)	Impulsivity	LHA	5 male and 11 female BPD high lethality suicide attempters; 5 male and 30 female BPD low lethality suicide attempters	Among low lethality suicide attempters, aggression was positively associated with gray matter volumes most robustly in the right insula (*k* = 166 voxels (35, 19, 9), *p* < 0.006) and bilateral fusiform gyrus (L *k* = 156 voxels (−35, −24, −19), *p* < 0.001, R *k* = 156 voxels, (37, −24, −19), *p* < 0.005). Impulsivity was negatively associated with gray matter volumes in nine ROIs among Low Lethality attempters, most widely in the right middle-superior temporal cortex *k* = 466 voxels (52, −48, −2), *p* < 0.001, bilateral insula (L *k* = 177 voxels (−35, 2, −1), *p* < 0.007; R *k* = 177 voxels (40, −3, 1), *p* < 0.002), and bilateral lingual gyrus (L *k* = 277 voxels (−17, −63, 0), *p* < 0.012; R *k* = 277 voxels (22, −88, −7), *p* < 0.0017).
Soloff et al. (2014b)	Impulsivity	LHA	20 female and 13 male BPD; 12 female and 15 male healthy controls	Among female BPD subjects, trait impulsiveness was inversely related to altanserin binding potential (BP_ND_) in medial frontal cortex (L beta (±s.e) = 128.3 (18.7), c.i. = 88.3, 168.3, *p* = 0.032; R beta (±s.e.) =133.8 (19.0), c.i. = 93.08, 174.42, *p* = 0.02), while aggression was negatively related to BP_ND_ in medial orbitofrontal cortex (L beta (±s.e.) = 36.4(8.8), c.i. = 17.6, 55.2, *p* = 0.009), and R beta (±s.e.) = 35.5(7.3), c.i. = 19.8, 51.1, *p* = 0.003). There were no significant relationships between these traits and BP_ND_ in male BPD subjects.
Wagner et al. [52]	Impulsivity	BPAQ	33 female BPD participants; 33 female healthy controls	BPD patients exhibited stronger functional connectivity from the noradrenergic locus (e.g., locus coeruleus) to the ACC, which was positively correlated with the degree of motor impulsiveness in the BPD sample (*t* = 5.35, *p* < 0.001, cluster size = 94, pFWE < 0.05). Furthermore, while controlling for aggression, stronger functional connectivity was detected between the serotonergic nucleus centralis superior (NCS) and the frontopolar cortex in patients versus controls (*t* = 4.87, *p* < 0.001, cluster size = 54, pFWE < 0.001).
Ende et al. [53]	Impulsivity	LHA	26 females with BPD; 22 females with ADHD; 30 female healthy controls	A significant positive correlation of glutamate to creatine ratio with impulsivity for the BPD participants was demonstrated (*r* = 0.32, *p* = 0.005). A negative correlation for the BPD participants for aggression with GABA was also found (*r* = 0.32, *p* = 0.005).
Kolla et al. [54]	Impulsivity	BDHI	11 males with ASPD; 5 males with ASPD and SCZ; 11 male healthy controls; 5 male SCZ patients without ASPD	In the ASPD group, there were negative correlations between dorsal caudate (*r* = −0.58, *p* = 0.023), dorsal putamen (*r* = − 0.55, *p* = 0.034), and cerebellum (*r* = −0.60, *p* = 0.017) [^11^C]CURB λ*k*_3_ with sensation-seeking impulsivity, controlling for genotype, and a significant negative association between cerebellum [^11^C]CURB λ*k*_3_ (*r* = −0.54, *p* = 0.035) and assaultive behavior, controlling for genotype.
Hosking et al. [56]	Risk Taking	Number of convicted crimes	49 adult male incarcerated offenders with a mean PCL-R of 24	Higher psychopathy (PCL-R score) was associated with stronger subjective value-related striatal activation (within the nucleus accumbens (NAcc)) during inter-temporal choice behavior (L *r* = 0.03, *p* = 0.846; R *r* = 0.335, *p* = 0.024) and with weaker cortico-striatal connectivity (*k* = 246 voxels, Z = 3.84, p_FDR_ = 0.017). Across all participants, both stronger striatal value-related activation (z = 0.295, *p* = 0.049) and attenuated cortico-striatal connectivity (*r* = −0.395, *p* = 0.009) were associated with a greater total of convicted crimes.
Prehn et al. [59]	Risk Taking	Factors of Aggressiveness	11 male hypo-reactive offenders with ASPD (high PCL-R factor 1 score and not more than 2 BPD criteria met; 12 male hyper-reactive offenders with ASPD (low PCL-R factor 1 score and diagnosis of BPD; 13 healthy male controls	Hypo-reactive offenders differed from healthy controls by exhibiting decreased neural activation in rostral ACC in response to uncertainty (z = 4.06, *p* < 0.001). There was a positive correlation in hypo-reactive offenders between right inferior frontal gyrus activity preceding a “stock” choice and an aggression measure as well as the number of risk-seeking mistakes (*r* = 0.42, *p* = 0.011).
Hofhansel et al. [55]	Callousness^a^	AQ, RPQ	27 male violent offenders; 27 healthy non-offender controls	PCL-R score was negatively correlated with prefrontal gray matter volume (*R*^2^ = 0.570, *p* < 0.001); this was primarily driven by the antisocial behavior (Facet 4) sub-scale of the PCL-R (*R*^2^ = 0.697, *p* < 0.001). There was also reduced GMV in right superior frontal (R^2^ = 0.677, *p* < 0.001), hippocampal (*R*^2^ = 0.548, *p* < 0.001) and left inferior parietal (*R*^2^ = 0.527, *p* < 0.001) regions with increasing antisocial behavior. One cluster comprising the right middle and superior temporal gyrus was negatively correlated with both reactive aggression (*R*^2^ = 0.408, *p* < 0.001) and antisocial (*R*^2^ = 0.623, *p* < 0.001) behavior.

*ASPD* Antisocial personality disorder, *AQ* Aggression Questionnaire, *BPAQ* Buss–Perry Aggression Questionnaire, *BPD* Borderline personality disorder, *LHA* Life History of Aggression, *OAS-M* Overt Aggression Scale-Modified, *PCL-R* Psychopathy Checklist-Revised, *PSAP* Point Subtraction Aggression Paradigm, *RPQ* Reactive-Proactive Aggression Questionnaire, *SCZ* Schizophrenia, *STAXI* State-Trait Anger Expression Inventory.

^a^This study examined correlations of PCL-R subscales (Facets) to neuroimaging findings: we considered Facet 2 PCL-R score as a proxy measure of trait callousness; potential link between Facet 4 to impulsivity also discussed in main text.

## Results

### Neuroimaging studies investigating anger/hostility and aggression

Several studies have examined the relationship between neural correlates of anger/hostility and aggression. We identified five studies including individuals with BPD and four studies with violent offenders with at least the majority having a personality disorder diagnosis – mostly ASPD.

In one study [30], 48 female patients with BPD and 28 healthy women participated in the Social Taylor Aggression Paradigm, a fMRI-compatible modification of the Taylor Aggression Paradigm. As in previous studies [31, 32], healthy women responded with higher activation in the amygdala and orbitofrontal cortex (OFC), as well as with higher aggression in trials with an angry versus neutral looking opponent. However, women with BPD did not show this emotion-dependent modulation. In both groups, there was a positive correlation between amygdala responsivity and aggression; however, in healthy women, this was for angry faces, while in women with BPD, it was for neutral faces. This suggests that, when in a context of provocation, women with BPD might not be able to adequately differentiate neutral/friendly from angry/hostile interpersonal signals, and this biased processing is even higher in those patients who react aggressively.

Another study [33] measured neural correlates of acting out in anger (a proxy marker of aggression) in 15 anger-prone men with BPD and 25 healthy men using an fMRI-compatible emotional approach avoidance task. Similar to a previous study with female BPD patients [34], men with BPD showed a tendency to approach rather than avoid angry faces and reduced ventrolateral prefrontal cortex (vlPFC) activations in incongruent trials, compared to healthy controls. In addition, the tendency to act out in anger (subscale of the State-Trait-Anger-Inventory; STAXI) was negatively related to vlPFC and dorsolateral prefrontal cortex (dlPFC) activation but positively related with amygdala activity in men with BPD. Since similar findings were previously reported in men with ASPD/psychopathy [35], such a deficit in fast emotional action control could be a common neural mechanism of anger-proneness and hostility predisposing an individual to aggressive reactions. A role for the vlPFC is supported by a further study [36], which demonstrated reduced gray matter volume in vlPFC in BPD patients with versus without a history of childhood abuse (the total sample included 18 individuals with BPD and 19 healthy controls). Intriguingly, gray matter in this region was related to both aggression and a form of hostility (“negativism,” as defined by the Buss-Durkee Hostility Inventory) in patients with childhood abuse. Although no sub-analysis of the link between aggression and hostility was performed in this sample, these findings suggest a possible interaction between structural vlPFC atypicality, trait hostility, and aggressive acts in men with BPD.

A further study [37] used a script-driven imagery paradigm to induce feelings of anger with standardized vignettes describing prototypical situations of interpersonal rejection, provocation, and frustration followed by aggressive reactions. Notably, this study included 23 male and 33 female anger-prone patients with BPD as well as 26 healthy men and 30 healthy women, allowing for analysis of sex by group interactions. Findings demonstrated that angerinduction led to increased amygdala activity in only men with BPD and not in healthy men and women with BPD. Male patients with BPD also showed elevated activations in the amygdala, OFC and dlPFC, while imagining anger-induced aggression. Furthermore, trait anger (STAXI subscale) was negatively associated with amygdala-dlPFC connectivity, while trait aggression (Buss–Perry Aggression Questionnaire; BPAQ) correlated positively with the amygdala-thalamus coupling in male patients.

Using fMRI, another group compared a sample of 25 healthy controls with 33 aggressive BPD patients (20 receiving specific anti-aggression group psychotherapy and 13 patients taking part in a non-specific control psychotherapy) [38]. Findings demonstrated a significant reduction in amygdala response and increased dorsomedial prefrontal cortex (dmPFC)-amygdala connectivity to emotional faces (including angry and fearful expressions) in an emotional face matching task in the aggressive patient group. Importantly, changes in amygdala activity and dmPFC-amygdala connectivity were related to changes in aggressive behavior (assessed with the Overt Aggression Scale-modified (OAS-M)) from pre- to post-treatment in the anti-aggression psychotherapy group only. These findings suggest the utility of targeted psychotherapeutic treatment approaches for subgroups of patients with BPD, based on level of aggression, which is in keeping with development of personalized medicine approaches.

Two studies, using an overlapping sample of violent male offenders, provided further insight into the link between neural correlates of anger/hostility and aggression in ASPD. In one [39], a correlation was demonstrated between a composite aggression measure and elevated amygdala reactivity to fearful faces in a similar emotional face matching task that was also reported in 19 incarcerated violent male offenders with personality disorders (mostly ASPD) compared to 28 healthy men. In the other [40], increased responsivity to provocation in the amygdala and striatum and a reduced connectivity between the ventromedial prefrontal cortex (vmPFC) and amygdala as well as striatum was found in 18 offenders, compared to 26 controls. Notably, vmPFC reactivity to provocation was positively related to trait anger (STAXI subscale) and aggression (BPAQ) across groups. In another study [41], violent offenders – the majority with a personality disorder diagnosis, mostly ASPD – were presented auto-taped thoughts and beliefs in response to angry, neutral, and happy situations and asked to either focus on their emotional feelings (engagement condition) or to regulate their feelings (distraction condition). Before and after this task, resting-state fMRI was performed. During anger engagement, increased vlPFC activation was found in 16 violent offenders compared to 18 non-offender controls, while the opposite (increased activity in offenders versus controls) was found in vlPFC and dlPFC during anger distraction [41]. Furthermore, reduced amygdala and vlPFC activity during anger distraction were positively related to self-reported aggression (BPAQ and Reactive Proactive Questionnaire). Analyzing resting state activity patterns in the same sample, a further study [42] found a positive correlation between anger (Anger-Single Target Implicit Association Test) and medial prefrontal cortex (mPFC) activity before the anger task, which increased during the task. Furthermore, the connectivity between mPFC and amygdala decreased in 18 violent offenders, while it increased in 18 controls during the task, and the opposite pattern was found for the connectivity between amygdala and (para)limbic regions.

Together, these studies suggest altered processing of anger-inducing situations in aggressive individuals. This seems to involve both more automatic, limbic reactions as well as prefrontal processes of cognitive control. However, the interplay of these regions, dynamic patterns, and the precise situational triggers remain unclear.

### Neuroimaging studies investigating impulsivity and aggression

Several studies of BPD and ASPD have examined the neural correlates of impulsivity and the relationship between impulsivity and aggression using imaging techniques. In general, neuroimaging investigations in BPD have reported structural, metabolic, and functional alterations of fronto-limbic networks that provides a neural basis for emotional dysregulation and impulsive and aggressive behavior [43–45]. One study of 31 female BPD participants and 25 female control subjects participated in an fMRI Go/No-Go task that presented negative (e.g., angry, sad, fearful), positive (e.g., happy), and neutral Ekman faces to elicit functional responding [46]. Trait impulsivity in BPD, measured using the Barratt Impulsiveness Scale-11 (BIS-11), was found to positively correlate with activation in the dorsal anterior cingulate cortex (dACC), OFC, dlPFC, and basal ganglia, while aggression negatively correlated with OFC, hippocampus, and basal ganglia activation. Negative emotional context and trait impulsivity, but not aggression, decreased task performance across groups. These results suggest that as an alternative to the “top-down, bottom-up” model proposed for affective interference with cognitive function in women with BPD [47, 48], negative emotion arising from situational stressors interrelates with the pre-existing neurobiology of personality traits, such as impulsivity, resulting in affective interference of neural processing of cognitive functions [46].

A different analysis of the same sample of 51 mixed sex BPD participants compared impulsivity (BIS-11), aggression (LHA), and brain structure using sMRI in high and low lethality suicide attempters [49]. No significant difference was noted between high and low lethality suicide attempters in terms of aggression or impulsivity. However, higher degrees of medical lethality were associated with decreased gray matter volumes across fronto-temporal-limbic regions, and effects of impulsivity and aggression on gray matter volumes differentiated high from low lethality attempters and differed within lethality groups. These results imply that lethality of suicide attempts in BPD could be related to mediation of aggression and impulsivity by specific neural networks.

The same group [50] used [^18^F]altanserin PET to quantify whether sex had a significant effect on the associations between 5-HT_2A_ binding; personality traits, such as impulsivity and aggression; and suicidal behavior in BPD. Thirty-three BPD patients (mixed sex) and 27 healthy controls (mixed sex), all unmedicated, were examined. The group had previously found effects of sex among healthy volunteers on the association between 5-HT_2A_ binding and aggression [51]. In the current study, among female BPD subjects, trait impulsiveness was inversely related to [^18^F]altanserin binding potential (BP_ND_) in medial frontal cortex, while aggression was negatively related to BP_ND_ in medial OFC. There were no significant relationships between these traits and BP_ND_ in male BPD subjects. Additionally, among BPD subjects, aggression, cluster B comorbidity, ASPD, and childhood abuse each related to altanserin binding. Therefore, region-specific differences in 5-HT_2A_ binding related to diagnosis, sex, and history of childhood abuse may relate to the clinical expression of aggression and impulsivity in BPD.

One resting-state investigation explored whether neurochemical systems, including the noradrenergic, dopaminergic, and serotonergic neurotransmitter systems, may be involved in the impulsivity of BPD [52]. This study evaluated the functional connectivity of the main monoamine-producing nuclei within the midbrain and brainstem in 33 unmedicated female participants with BPD and 33 matched healthy controls to relate any altered functioning of these nuclei to the patient’s impulsivity. Although multiple regression did not detect any significant association between impulsivity and altered functional connectivities in the BPD group, BPD patients exhibited stronger functional connectivity from the noradrenergic locus (e.g., locus coeruleus) to the ACC, which was positively correlated with the degree of motor impulsiveness in the BPD sample. Furthermore, while controlling for aggression, stronger functional connectivity was detected between the serotonergic nucleus centralis superior (NCS) and the frontopolar cortex in patients versus controls. While the fMRI modality utilized in the current study cannot directly implicate dysfunction of monoamine neurotransmission in BPD, enhanced locus coeruleus-ACC resting state functional connectivity in women with BPD and its link to motor impulsiveness could indicate noradrenergic dysfunction in neural inhibitory control networks, while increased NCS-frontal pole resting state functional connectivity could implicate serotonergic signaling in prefrontal control of aggressive behavior.

An investigation that sampled 26 females with BPD, 22 females with attention deficit hyperactivity disorder (ADHD), and 30 female healthy controls also considered neurochemical underpinnings. This study explored the relationship between measures of impulsivity and aggression and ACC glutamate to total creatine ratios (Glu/tCr) and GABA levels using single voxel 1H magnetic resonance spectroscopy [53]. Self-rating scales, including the BIS-11 and Brown Goodwin Lifetime History of Aggression (BGLHA), to evaluate impulsivity and aggression, respectively, were employed. When analyses were parsed by individual diagnoses, group-wise correlational analyses yielded a significant positive correlation of Glu/tCr with BIS-11 total score for the BPD participants and a negative correlation for the BPD and the healthy control participants for the BGLHA aggression score with GABA. However, neither correlation was significant for the ADHD group. These results provide some evidence for the role of excitatory and inhibitory neurotransmitters in the pathology of impulsivity and aggression in women with BPD.

An [^11^C]CURB PET study [54] that investigated fatty acid amide hydrolase (FAAH), an enzyme of the endocannabinoid system that degrades anandamide and thereby indirectly regulates cannabinoid receptor signaling, examined 16 males with ASPD and 16 male control participants (five with schizophrenia). Results revealed that cerebellar and striatal FAAH expression were inversely related with impulsivity, while cerebellar FAAH density was also negatively associated with assaultive aggressive. These results point to a potential endocannabinoid-lowering process in ASPD that could affect manifestation of impulsivity and aggression in this population.

Finally, one study demonstrated that in 27 violent offenders, gray matter volume in multiple prefrontal regions including superior frontal gyrus and superior orbital gyrus was negatively associated with PCL-R Factor 2 traits [55]. However, further analysis revealed that this effect was mostly driven by Facet 4 traits (antisocial behavior), rather than Facet 3 traits (which includes impulsivity). This study is discussed in further detail in the section on callousness (below).

Taken together, these studies suggest that abnormalities of fronto-temporal-limbic regions are implicated in the impulsivity of BPD and ASPD and may predispose to aggressive behaviors. Neurochemically, alterations in serotonergic and endocannabinoid system signaling pathways may also give rise to impulsivity and aggression in these populations. It should be noted that most of the reviewed studies of impulsivity and aggression in BPD and ASPD used questionnaire-based measures of impulsivity, as opposed to neuropsychological paradigms, to assay these constructs.

### Neuroimaging studies investigating risk-taking and aggression

We found two studies linking neural correlates of risk-taking to metrics of aggression, both in incarcerated male offenders. In one of study [56], 49 adult male incarcerated offenders with a mean PCL-R score of 23.5 were administered an intertemporal choice (e.g., delay-discounting) task, while using a mobile fMRI scanner to investigate task-related activation and resting-state functional connectivity. Higher psychopathy (PCL-R score) was associated with stronger subjective value-related striatal activation (within the nucleus accumbens [NAcc]) during inter-temporal choice behavior and with weaker cortico-striatal connectivity (between NAcc and ventromedial prefrontal cortex [vmPFC]), suggesting a potential link between these abnormalities and risky decision-making in personality-disordered men. Further, across all participants, both stronger striatal value-related activation and attenuated cortico-striatal connectivity were associated with a greater total number of convicted crimes. These results suggest that dysregulated cortico-striatal circuits may drive risky decision making across a spectrum of antisociality in men and underscore value-based decision-making as a potential proximal mechanism underlying self-control deficits in disinhibitory syndromes [57, 58].

Another study of violent criminal offenders explored emotion-related mechanisms leading to risky decisions using an fMRI paradigm, where respondents were required to choose between low-risk bonds and high-risk alternatives, such as stocks [59]. While bonds were always a safe choice, stocks could win or lose with varying certainty. All of the offenders met criteria for ASPD. This group was further subdivided into emotionally hypo-reactive offenders (e.g., high PCL-R Factor 1 score and not more than two BPD criteria met; *n* = 11) and hyper-reactive offenders (e.g., low PCL-R Factor 1 and diagnosis of BPD; *n* = 12). Thirteen male healthy controls without a criminal or psychiatric history also participated. Results revealed that hypo-reactive offenders differed from healthy controls by exhibiting decreased neural activation in rostral ACC in response to uncertainty and decreased activity in the prefrontal cortex when consistently choosing safe alternatives. There was a positive correlation in hypo-reactive offenders between right inferior frontal gyrus activity preceding a “stock” choice and subscale scores on a questionnaire measuring aggression as well as the number of risk-seeking mistakes, which was interpreted as a measure of behavioral dyscontrol.

### Neuroimaging studies investigating callousness/lack of empathy and aggression

One study in 27 violent offenders and 27 healthy controls [55] linked structural brain abnormalities in the offending group to both antisocial traits (PCL-R facets) and to aggression (using Aggression Questionnaire, AQ, and Reactive–Proactive–Aggression Questionnaire, RPQ) [60]. Total and sub-scale scores of these measures were correlated with gray matter volumes (GMVs), using VBM-based brain morphometry, in the offenders. For PCL-R scores, as noted in “impulsivity” section above, findings demonstrated a link specifically between prefrontal GMV and Facet 4 antisocial behavior traits, such as juvenile delinquency and recidivism. For Facet 2 traits, which include “callousness/lack of empathy,” there was no correlation. For aggression scores, only one sub-scale of the trait aggression scales correlated significantly with GMV in offenders. Specifically, RPQ reactive aggression was negatively linked with GMV in the right middle and superior temporal gyrus. Together, these findings suggest a link between prefrontal GMV and antisocial behavior, potentially mediated through reactive aggression. They do not provide support for the contribution of any GMV deficits and callousness/lack of empathy, which has typically been linked to proactive aggression. This is discussed further in the limitations section below. We did not find any further studies in our included subject groups that linked a neuroimaging metric to aggression and also to callous-unemotional traits or lack of empathy.

## Discussion

To explore how trait-based approaches to neuroimaging research in BPD and ASPD have progressed since publication of DSM-5, we conducted a systematic review of neuroimaging studies investigating key traits linked to aggression across these disorders. While a lack of methodological consistency in the field remains and limits the scope of our findings, our study identified some important considerations for future work.

First, evidence from studies identified in our review suggests that anger/hostility associated with alterations in the interplay between prefrontal (dlPFC, vlPFC, OFC, and mPFC) and subcortical regions (primarily the amygdala) could be a common factor explaining aggressive reactions in response to perceived interpersonal threat or provocation. Interestingly, findings indicate that a proneness to act out aggressively may be linked to a reduced differentiation (at a neural and behavioral level) between threatening and non-threatening interpersonal cues, in line with the hypothesis of a hostile filter that biases the perception of the entire social environment, thus increasing the likelihood for aggressive encounters. This is also in line with the findings of an earlier PET study, which revealed stronger amygdala responses to high as well as low provocation in individuals with comorbid BPD and IED [61]. However, this hypothesis needs to be tested in a large group of individuals across specific personality disorders and both sexes.

Second, there remains an overall lack of clarity about the respective links between neural correlates of impulsivity and aggression. This is surprising, given prior evidence suggesting that impulsive behavior and reactive aggression may share common neural underpinnings [62]. One potential explanation is that in the majority of studies linking impulsivity to aggression in this review, questionnaire-based measures of impulsivity were employed. This may be critical, because neuropsychological testing of impulsivity does not uniformly overlap with impulsivity measured via self-report [52]. In fact, some authors argue that data are lacking for a relationship between behavioral impulsivity and self-reported impulsivity, possibility pointing to different constructs [63]. Whether a lack of neuropsychological measures testing impulsivity in these studies has relevance for understanding the neural correlates of aggression in BPD and ASPD is currently unknown. However, from the reviewed studies, alterations in the structure and function of fronto-temporal-limbic regions are implicated in the impulsivity of BPD and ASPD that may give rise to aggressive behaviors. Furthermore, there may be a role for certain neuromodulatory systems, such as the serotonergic or endocannabinoid signaling systems, in connecting impulsive behavior to aggressive responding. This is also consistent with the results of a row of earlier studies in individuals with IED, which revealed a role for the serotonergic system in impulsive aggression [64, 65].

Another potential explanation for this lack of clarity is the conceptual overlap between impulsivity and risk taking. This may explain in part why our search yielded so few studies specifically examining links between risk-taking and aggression. Whereas impulsivity may reflect acting on the spur of the moment in response to immediate stimuli, acting on a momentary basis without consideration of outcomes, or difficulty establishing and following plans, risk-taking may involve poor representation of the degree of risk, especially in situations where the degree of risk gradually increases combined with the magnitude of a potential favorable outcome when the precise odds of negative outcomes are unknown or not explicit. However, some imaging reports in healthy populations have argued for a clear dissociation between high-risk behavior tendency as a construct distinct from that of impulsivity [66]. We propose that a clearer demarcation in the AMPD system between impulsivity and risk-taking could shed further light on possible unique neural correlates between these traits and aggression in BPD and ASPD.

Third, we found a notable lack of studies that investigated potential neuroimaging correlates for both callousness/lack of empathy and aggression. This was also surprising, considering considerable previous evidence suggesting a neurobiological component to callousness [67–69] and the putative link between callousness and aggression, particularly proactive aggression [29]. While a detailed neurobiological model of reactive aggression has been previously outlined [70], a corresponding model for proactive aggression has not yet emerged. However, the integrated emotion system (IES) model [71] offers one potential explanation, in those high on trait callousness/lack of empathy. First, reduced amygdala functioning, which leads to impaired processing of fear, may result in a lack of deterrence from harming others to gain advantage. Second, decision-making deficits driven by striatal dysfunction and other reward-related circuitry such as vmPFC [72] may lead to those high on trait callousness/lack of empathy to take pleasure in causing harm to others. These deficits are seen in individuals with ASPD and psychopathy, in whom callousness/lack of empathy and proactive aggression is characteristic [29, 73]. However, there is some evidence that callousness may also play a role in reactive aggression. One PET study with patients with IED characterized by high levels of impulsive aggressive outbursts found trait callousness exhibiting a significant positive correlation with the serotonin transporter availability in the ACC [74]. Future work will benefit from examination of these potential neurobiological underpinnings of aggression, for example, by linking performance on empathy-inducing fMRI tasks with behavioral measures of reactive and proactive aggression.

Our review identified further gaps in the existing literature. First, the included studies used a variety of different measures for aggression, ranging from different self-report trait questionnaires, interviews assessing aggressive behavior within the past weeks, to aggressive responses in experiments. Although acceptable reliability has been shown for most of these measures, their ecological validity remains questionable. There are several reasons for this: i) the correlations between different measures of aggression are often small to moderate; ii) most of these measures are subject to social desirability effects; iii) measuring aggression in a highly standardized yet ecologically valid experiment is a particular challenge in a neuroimaging setting; and iv) most of these measures do not provide information about which particular situation a particular person acts out aggressively in real life. Hence, future studies combining neuroimaging methods with ecological momentary assessment are needed.

Second, there was a marked lack of specificity about forms of aggression in most studies in our review. This is especially important as previous evidence suggests that reactive aggression is more likely be associated with anger and impulsivity [62] and proactive (e.g., instrumental) aggression may be distinctly linked to severity of callousness/lack of empathy [75, 76]. Arguably, new paradigms are required that are better able to differentiate between these subtypes of aggression and also between different triggers for aggression (e.g., provocation, frustration, threat).

Third, none of the studies in our review investigated more than one of the personality traits potentially related to aggression within a single sample. This limits inferences on the specificity of findings from individual studies. It also precludes investigation of the interplay between these specific traits in a causative model of aggression. Future studies will benefit from a principled approach to exploring links between the relative contributions of individual traits (selected based on prior work demonstrating links to aggression) - and their neural underpinnings - to aggression. Analytical models that can explore whether particular traits, or combinations of traits, mediate the correlation between neural signatures of aggression, or vice versa, would be particularly beneficial. Such studies will likely require more specific measures of aggression, as well as large and heterogeneous samples of aggressive individuals with personality disorders [13].

Fourth, with few exceptions, the majority of neuroimaging studies of aggression in BPD samples involve women only, while neuroimaging studies of aggression in ASPD and psychopathy focus on men. To some extent, these patterns reflect the prevalence of each condition by sex under certain scenarios. For example, females with BPD are over-represented in clinical settings [77], while ASPD is 5–7 more times common in males than females [78]. Still, it cannot be assumed that the neurobiology of aggression in ASPD males is the same as in females, or conversely, that neuroimaging findings in female BPD patients are the same as in male BPD subjects [37]. A more fulsome understanding of sex differences and their underlying neurobiology may be important in developing sex-specific treatment programmes. For example, limited research suggests that males with BPD experience a greater reduction in physical aggression and develop enhanced anger management skills compared to females with BPD in a dialectical behavior therapy program specifically modified for corrections [79].

### Future directions

Previous reviews of clinical studies using AMPD have suggested that this model demonstrates acceptable interrater reliability, largely consistent latent structures, substantial convergence with relevant external measures, evidence for incremental validity when controlling for categorical PD diagnoses [80], and clinical utility [81]. Our review has highlighted that applying a trait-based approach to the neurocognitive basis of personality disorders may additionally yield mechanistic insights. However, future work should acknowledge potential limitations of this approach. Other studies have demonstrated high correlation of criterion A and B [80], correlation of criterion A with both axis I and axis II disorders [82], and the finding that traits (criterion B) account for substantially more unique variance in DSM-5 Section II PDs than does personality impairment (criterion A) [82, 83]. Moreover, many clinicians and researchers continue to have reservations about several aspects of application of AMPD, including the communicative value between clinicians and their patients’ families, the feasibility of the model’s application, and the model’s ability to translate into treatment modalities [81]. Future studies should address these issues and seek to demonstrate added value over categorical approaches, in order to justify a wider shift to the dimensional approach of AMPD.

Furthermore, some consideration should be given to the potentially loaded and negatively-valenced descriptors of certain personality traits. Consistent terminology about antisocial symptom domains remains important and has utility in inter-professional communication [84]. However, a shift to routine use of the terms “callousness” and “hostility”, especially in younger populations, may be deemed unacceptable by patient groups and their advocates. Notably, DSM-5 uses the alternate term “Limited Prosocial Emotions,” in specifier criteria for conduct disorder (the precursor of ASPD). Careful development and application of terminology will likely have a role in developing a consensus-based empirical approach in this area. Additionally, an overly reductionist portrayal of these symptoms as discrete entities, emerging from distinct neurobiological deficits, and which are relatively immutable, is likely to be misguided. For instance, emerging evidence in youth populations suggests that callous-unemotional traits are themselves heterogeneous [85, 86] and vary throughout personality development [87, 88]. Longitudinal neuroimaging data that illustrate consistent patterns of disrupted brain development will be an important further development [89].

Methodological issues also warrant further consideration. The lack of a consistent approach to inclusion criteria and stratification of groups of antisocial and violent offenders in studies could be addressed by consensus approach to defining both categorical measurements (e.g., DSM-5 criteria for ASPD and PCL-R for psychopathy) and symptom domains (e.g., using DSM-5 criteria for Limited Prosocial Emotions). Second, since aggression is a complex phenomenon, future studies will need to include multi-methodological designs: hormones and physiological measures indicating the level of peripheral arousal should accompany neuroimaging and self-reports. Several studies have suggested that arousal, for example, resting state heart rate [90] shows a moderate negative correlation with violent behavior. Similarly, associations between testosterone and cortisol levels and responses to stress or provocation and aggression need to be taken into account. Other methods besides neuroimaging, such as electroencephalography, may provide important insights in the timing of cortical processing and the level of automacy of the above discussed processes. Ecological momentary assessments might be useful to acquire information about the situations in which an individual acts out aggressively and how this is related to maladaptive trait profiles.

## Conclusions

This systematic review examined trait-based approaches to aggression in neuroimaging research in BPD and ASPD published since the introduction of AMPD. While there were relatively few neuroimaging studies examining AMPD traits relevant to aggression in BPD and ASPD, several key themes emerged. First, a variety of different measures for aggression exist, but studies combining neuroimaging methods with ecological momentary assessment are needed to better understand under what situations particular individuals act out aggressively in real life. Second, very few of the studies differentiate between proactive and reactive forms of aggression, which has relevance for understanding how subtypes of aggression relate to AMPD traits. Third, the existing neuroimaging studies are limited to the study of only one particular trait in relation to aggression, when in reality most individuals will likely endorse multiple AMPD traits. Fourth, most studies of BPD focus on females, while those of ASPD sample males. This lack of heterogeneity makes it difficult to parse the neuroimaging markers of aggression in male BPD patients and female ASPD subjects. We have also highlighted methodological inconsistencies across the existing literature and emphasized the importance of a consistent approach to categorical and trait specification. We conclude that multi-methodological designs incorporating a range of biomarkers hold the most promise for understanding how a relatively new maladaptive trait model of personality disorders can better inform on the biological underpinnings of aggression in BPD and ASPD.
